# Bioinformatic Analysis of Kynurenine Pathway Enzymes and Their Relationship with Glioma Hallmarks

**DOI:** 10.3390/metabo12111054

**Published:** 2022-11-02

**Authors:** Gustavo Ignacio Vázquez Cervantes, Javier Ángel Navarro Cossio, Gonzalo Pérez de la Cruz, Aleli Salazar, Verónica Pérez de la Cruz, Benjamin Pineda

**Affiliations:** 1 Neurobiochemistry and Behavior Laboratory, National Institute of Neurology and Neurosurgery “Manuel Velasco Suárez”, Mexico City 14269, Mexico; 2 Department of Mathematics, Faculty of Sciences, Universidad Nacional Autónoma de México, UNAM, Mexico City 04510, Mexico; 3 Neuroimmunology Unit, National Institute of Neurology and Neurosurgery “Manuel Velasco Suárez”, Mexico City 14269, Mexico

**Keywords:** tryptophan catabolism, glioma, immune response

## Abstract

Indoleamine dioxygenase (IDO), a rate limiting enzyme of the tryptophan catabolism through the kynurenine pathway (KP), has been related with a lower survival and a poor patient prognosis on several solid tumors, including gliomas. However, the use of IDO inhibitors as a therapeutic strategy for tumor treatment remains controversial in clinical trials and the role of other KP enzymes on tumor progression has remained poorly understood so far. Recently, different studies on different types of cancer have pointed out the importance of KP enzymes downstream IDO. Because of this, we conducted a bioinformatic analysis of the expression of different KP enzymes and their correlation with the gene expression of molecules related to the hallmarks of cancer in transcriptomic datasets from patients with different types of brain tumors including low grade gliomas, glioblastoma multiforme, neuroblastoma, and paraganglioma and pheochromocytoma. We found that KP enzymes that drive to NAD+ synthesis are overexpressed on different brain tumors compared to brain cortex data. Moreover, these enzymes presented positive correlations with the expression of genes related to immune response modulation, angiogenesis, Signal Transducer and Activator of Transcription (STAT) signaling, and Rho GTPase expression. These correlations suggest the relevance of the expression of the KP enzymes in brain tumor pathogenesis.

## 1. Introduction

Astrocytomas are primary brain tumors originating from astrocytes [1]. Classically, astrocytomas had been classified according to their morphological characteristics and tumor aggressiveness into four stages [1,2,3,4]. However, the most recent WHO classification of astrocytomas considers molecular characteristics that are representative within astrocytomas and this new classification of astrocytomas separates these tumors according to the status of enzyme isocitrate dehydrogenase (IDH): wildtype (IDHwt) or mutant (IDHmt) [2].

Glioblastoma (GBM) is the most frequent and aggressive of the primary brain tumors [3]. Considering the current standard of care treatment for GBM, the median overall survival is no longer than 18 months, while the five-year survival is less than 10%, thus GBM is considered an incurable illness [3,4,5].

In the recent years, the study of cancer biology has been focused on a set of networking characters that led malignant cells to adopt a niche and to transform a heterogeneous landscape, such as that occurring during GBM formation; such characters are better known as the “Hallmarks of cancer” [6,7]. Within these hallmarks, genomic instability, metabolic reprogramming, and immune modulation are highly important. Genomic instability processes are responsible for originating somatic mutations on the malignant cell genome that drive cell to death evasion, cell cycle alteration, and metabolic reprogramming [6,7,8]. In this regard, according to mutational and transcriptional patterns, genomic subclassifications for GBM have been proposed [9,10].

On the other hand, metabolic reprogramming includes modifications on the regulation of several metabolic pathways that provide the malignant cells of biomolecules required to satisfy their bioenergetic and proliferative demands [6,11]. It has been widely accepted that the main metabolic transformation in GBM cells involves an exacerbated glycolytic activity through the production of lactate even under normoxia conditions (Warburg effect) [12]. However, recent evidence proposes that oxygen and nutrient availability as well as the stemness condition of the malignant cells produces a metabolic heterogeneity within the tumor tissue, making possible the presence of malignant cell populations with different metabolic signatures [13,14,15,16,17,18].

Furthermore, GBM cells elicit a set of processes that allow them to modulate the immune response, shifting it from an anti-tumor profile to a tumor-associated response [19]. GBM cells can produce anti-inflammatory cytokines such as transforming growth factor beta (TGB-β), the vascular endothelial growth factor (VEGF), interleukin (IL) 6, and IL-10, as well as to express cell surface inhibitory molecules like the cytotoxic T lymphocyte antigen-4 (CTLA-4) and programmed cell death-1 (PD-1) [19]. These mechanisms provoke GBM tissue to become highly infiltrated with tumor-associated macrophages (TAMs), myeloid-derived suppressor cells (MDSCs), and regulatory T cells (Tregs) which support tumor growth and invasiveness through the secretion of growth factors and matrix metalloproteinases [20,21]; while effector immune populations such as natural killer cells (NK) and cytotoxic lymphocytes remain anergic, unable to elicit anti-tumor responses [20,22]. Altogether, the immunomodulatory mechanisms developed by GBM represent a barrier for GBM treatment success [22].

Some of the altered processes present on GBM may contribute to more than one of the hallmarks of cancer, thus these shared processes could become promissory targets for the development of new therapeutic strategies. One of such processes that is taking importance is the tryptophan catabolism through the kynurenine pathway (KP). The KP is the main source of tryptophan degradation on mammals [23,24]. The KP activity is mainly limited by the enzymes tryptophan dioxygenase (TDO) and indoleamine dioxygenase (IDO); both enzymes produce N-formylkynurenine from the cleavage of tryptophan in hepatic and extrahepatic tissue, respectively [23,24]. N-formylkynurenine is rapidly converted into kynurenine (KYN), which can be taken by kynurenine aminotransferase (KAT), kynureninase (KYNU), and kynurenine monooxygenase (KMO) to produce kynurenic acid (KYNA), anthranilic acid (ANA), and 3-hydroxykynurenine (3-HK), respectively [23,24]. Then, 3-hydroxyanthranilic acid (3-HANA) is produced and leads to a picolinic and quinolinic acid formation [23,24]. Finally, quinolinic acid phosphoribosyl transferase (QPRT) produces the coenzyme NAD+ de novo [24,25]. The KP intermediate metabolites have been shown to produce deleterious effects on cytotoxic immune populations [26,27,28]. Moreover, IDO and TDO overexpression have been associated with GBM immunosuppression and a poor patient prognosis [29,30,31]. Furthermore, the expression of KMO on GBM and other solid tumors such as melanoma, mammary, and colorectal cancer has been recently reported, pointing to KMO as a promissory subject of study [32,33,34,35]. However, the role of KP enzymes on tumor cell biology and their relationship with the hallmarks of cancer needs to be better understood, which could help to locate the KP as a potential target for the development of therapeutic strategies for GBM.

In this study, we compared the expression profiles of the KP enzymes among GBM and low grade astrocytomas, according to the WHO classification and to the genomic subclassification of GBM using tumor bulk transcriptional data from public databases. We also searched for a correlation between the expression of KP enzymes and the gene expression of molecules associated with the hallmarks of cancer.

## 2. Materials and Methods

### 2.1. Data Acquisition and Sample Selection

Gene expression RNAseq data (RSEM norm_count) from a combined cohort of TCGA, TARGET, and GTEx samples available on the Xena platform [36]. From a total of 19,131 samples, only those corresponding to low-grade glioma (TCGA brain low grade glioma, *n* = 523), glioblastoma multiforme (TCGA glioblastoma multiforme, *n* = 166), brain cortex (GTEx brain cortex, *n* = 105; GTEx brain anterior cingulate cortex (Ba24), *n* = 83 and GTEx brain frontal cortex (Ba9), *n* = 95), pheochromocytomas and paragangliomas (P&P, *n* = 185), and neuroblastoma (NB, *n* = 162) were considered. Then, the IDH-1 somatic mutations were observed on low-grade glioma and glioblastoma multiforme samples; the samples without the IDH-1 status information were removed (*n* = 36).

Their gene expression was studied in detail naïven three groups: TCGA brain low-grade glioma with IDH-1 mutation (LLG IDH-m; *n* = 390), TCGA brain low-grade glioma with IDH-1 wildtype (LLG IDH-w, *n* = 114), and TCGA glioblastoma multiforme with IDH-1w (GBM, *n* = 142).

### 2.2. Selection of Gene Pools and Gene Expression Comparison

The first group of genes corresponds to the most important enzymes of KP: IDO1, KMO, KYNU, HAAO, AADAT, and GOT2. Then, five more groups were made based on some of the hallmarks of cancer and their representative genes: the immune response (HLA-E, HLA-G, CD274, PDCD1LG2, CTLA4, TIGIT, HAVCR2, LAG3, IFNG, TNF, TGFB1, IL1B, IL6, IL10, IL13, IL13RA2, and IL2RA), the angiogenesis (VEGFA, VEGFB, VEGFC, MMP2, MMP9, FGF1, FGF2, FGFR1, HGF, EGFR, and PTEN), the STATs (STAT1, STAT2, STAT3, and STAT4), the Rho GTPases (CDC42, RAC1, and RHOA), and the electron transport chain complex 1 and 2 (MT-ND1, NDUFS1, SDHA, and SDHB) (Table 1).

### 2.3. Statistical Analysis

For each of the gene expressions corresponding to the most important KP enzymes, the comparison between the groups of samples were performed using the Kruskal–Wallis test with Dunn’s test for multiple pairwise comparisons against non-tumor brain cortex samples. *p*-values < 0.05 were considered statistically significant. For each group of samples, the pairwise correlations among the expressions of each of the genes corresponding to the most important enzymes of KP and each of the genes of the other five groups of genes were assessed using the Spearman’s correlations with the Holm adjustment for multiple tests. *p*-values < 0.05 were considered statistically significant. To assess the effect of the KP enzymes in the survival probability, for each KP enzyme, a Cox proportional hazards regression model with a stratification defined by the three groups of samples (LGG-IDHm, LGG-IDHw, and GBM-IDHw), with one continuous covariate (the expression levels of the enzyme), and including the interaction between this covariate and the groups of samples, was adjusted. With this model, three tests were performed to assess the global effect of the enzyme on the survival probabilities, that is, that there is an effect in at least one of the groups of samples: the likelihood ratio test and Wald test and score (logrank) test. Then, tests with adjustments for multiple comparisons were performed to identify the group of samples where the effect is significant. The numerical results were obtained using R v4.0.2 (Vienna, Austria) [37], mainly packages survival (v3.1-12) [38], multcomp (v1.4-15) [39], and psych (v2.0.9) [40].

## 3. Results

### 3.1. KP Enzymes Distribution on Brain Tumors

**Figure 1:**
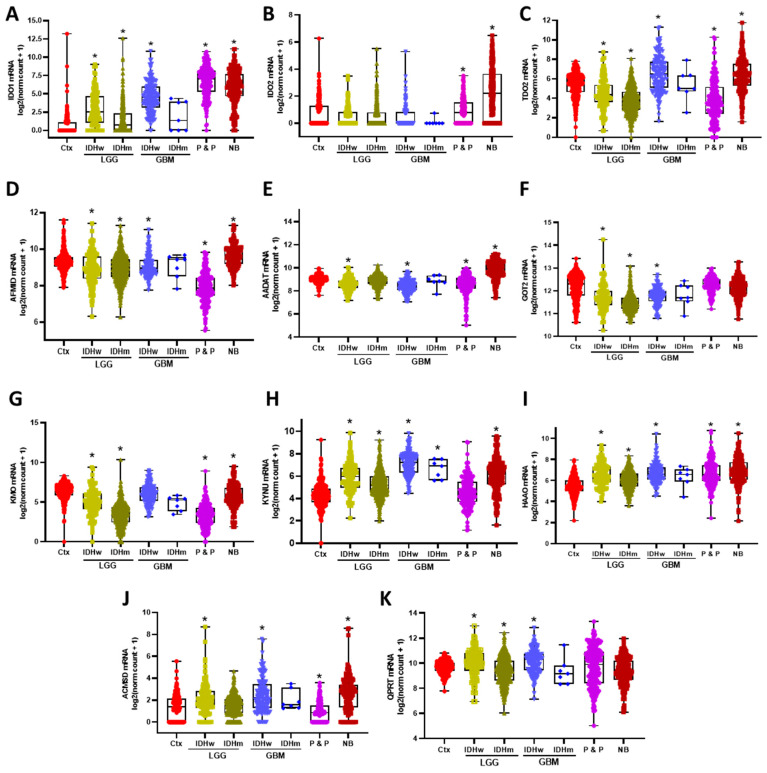
Differential gene expression of the KP enzymes among brain tumors. Low grade gliomas (LGG) and glioblastoma (GBM) were separated according to their IDH wildtype (IDHw) or IDH mutant (IDHm) status, then expression levels of the KP enzymes IDO1 (**A**), IDO2 (**B**), TDO2 (**C**), AFMID (**D**), AADATnaïve (**E**), GOT2 (**F**), KMO (**G**), KYNU (**H**), HAAO (**I**), ACMSD (**J**), and QPRT (**K**) were compared with non-tumor brain cortex samples (Ctx), pheochromocytomas and paragangliomas (P & P), and neuroblastomas (NB). * *p* < 0.05 when considering the Dunn’s test for multiple pairwise comparisons against Ctx.

First, the expression of the KP enzymes on different brain tumors was explored, considering that LGG and GBM were separated according to their IDH mutational status. Under these conditions, it was found that among the tryptophan degrading enzymes (IDO1, IDO2, and TDO2), the IDO1 expression was significantly higher in all different types of tumors compared to the brain cortex, except in IDHm GBM, although the exception could be due to the sample size (Figure 1A). Unlike gliomas, both the paraganglioma and pheochromocytoma and the neuroblastoma groups presented an increased expression of IDO2 (Figure 1B), while IDHw GBM and neuroblastomas presented a higher expression of TDO2 compared to the brain cortex. IDHw LGG and IDHm LGG and P&P presented a decreased expression of TDO2 compared to the brain cortex (Figure 1C). On the other hand, AFMID, the enzyme leading to the formation of KYN, showed a decreased expression in all the different tumors compared to the brain cortex, except in neuroblastoma (Figure 1D). Additionally, the AADAT and GOT2, the KAT’s enzymes that lead to a KYNA formation, presented a decreased expression among the different tumors compared to the brain cortex, except in the neuroblastoma group, in which the expression of AADAT is higher compared to the brain cortex (Figure 1E,F).

KMO, the other enzyme that can take KYN as a substrate and produce 3-HK, showed a decreased expression in all CNS tumors which were evaluated compared to the brain cortex levels (Figure 1G). Contrastingly, the expression of the KP enzymes that lead to NAD+ synthesis, KYNU, HAAO, ACMSD, and QPRT were significantly increased in brain tumors compared to the brain cortex (Figure 1H–K). It is of note that there were differences within IDHw LGG and GBM compared to their IDHm counterparts in IDO1, TDO2, KMO, and QPRT expressions.

### 3.2. Correlation between KP Enzymes and Glioma Hallmarks

**Figure 2:**
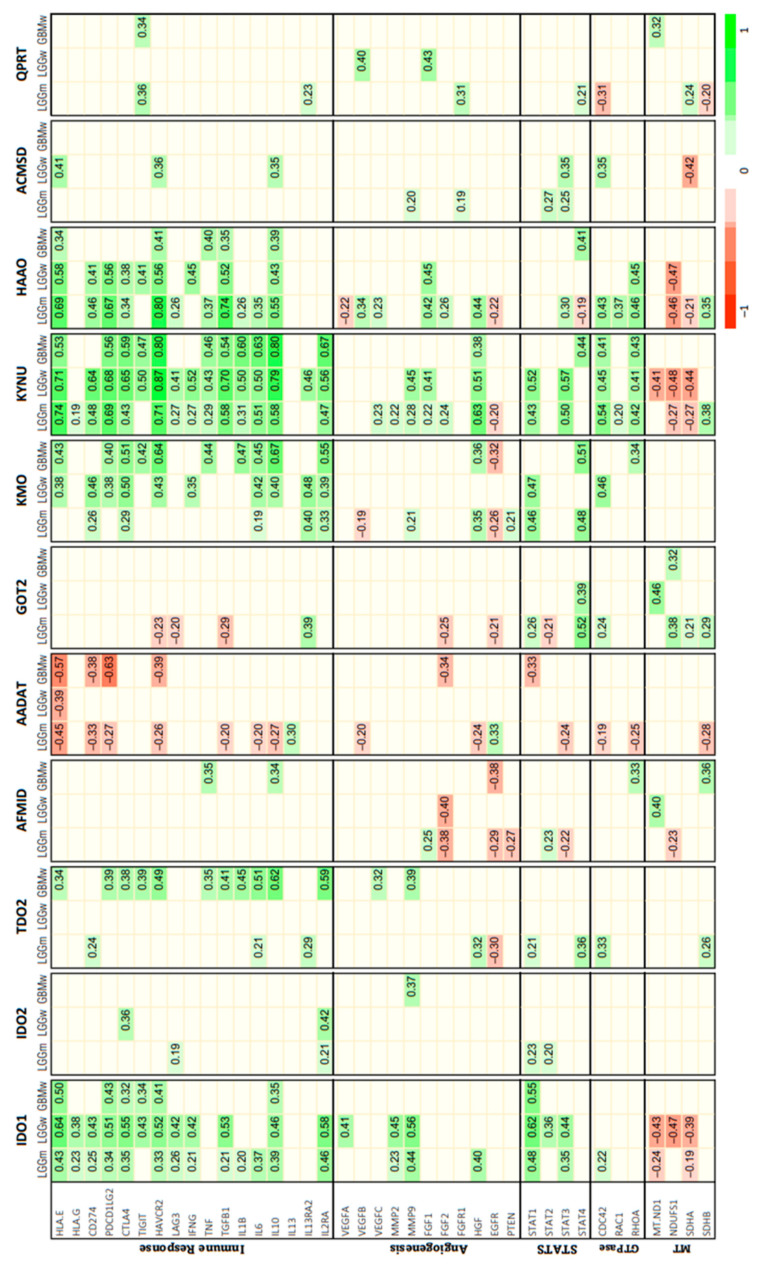
Heat map of Spearman’s correlations between the kynurenine pathway expression and diverse glioma hallmarks. Only the significant correlations when considering the Holm adjustment for multiple tests in each group of samples are presented.

After describing the distributions of the KP enzymes on the CNS tumors, we looked for a correlation between the KP enzymes and the gene expression of molecules associated with the hallmarks of cancer (Figure 2). In this way, it was found that the gene set with more correlations with KP enzymes was the ‘immune response’ set, being KYNU, IDO, HAAO, and KMO, the enzymes which presented more positive correlations with immune checkpoint molecules such as HLA-E, PDCD1LG2 (PD-L1), CTLA-4, HAVCR2 (TIM3), and interleukins 6 and 10. It is of note that KYNU presented significant correlations with genes from other hallmark sets like angiogenesis, STAT signaling, small Rho GTPases, and mitochondrial genes.

Interestingly, AADAT expression presented negative correlations with the genes studied here, mainly the genes related with the immune response. GOT2 also shows negative correlations but with fewer genes.

Regarding IDO2, TDO2, AFMID, ACSMD, and QPRT present only some correlations, with a few genes of those of the different hallmark sets.

### 3.3. Impact of KP Enzymes on Survival Estimation

**Figure 3:**
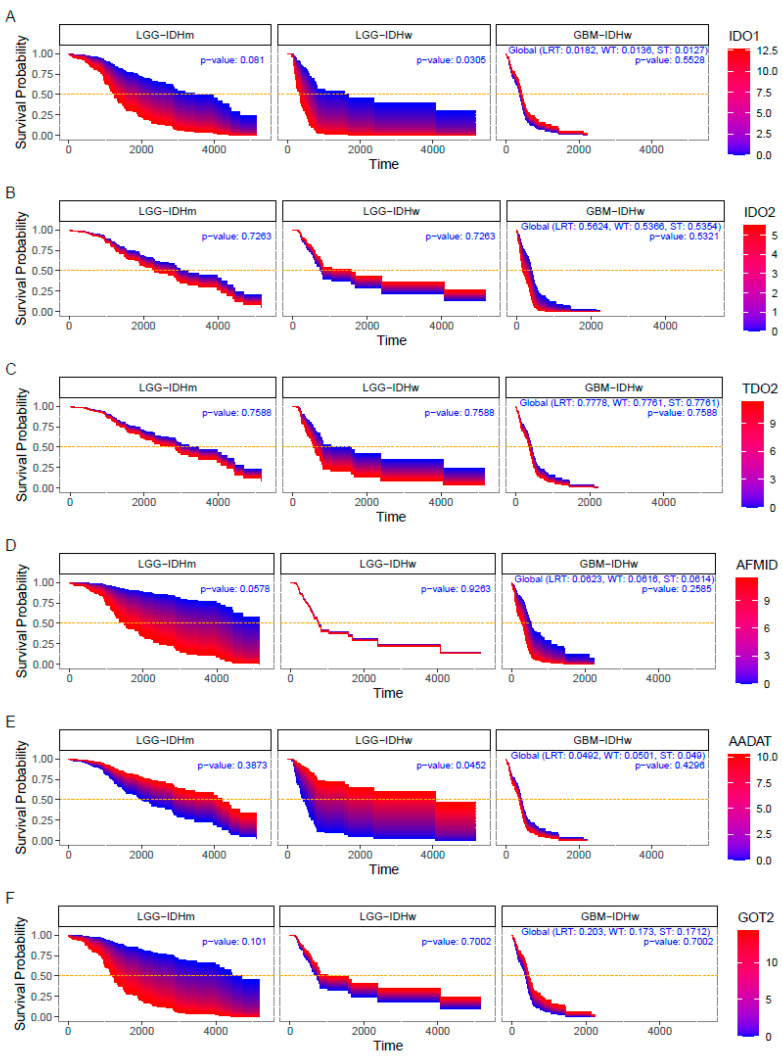

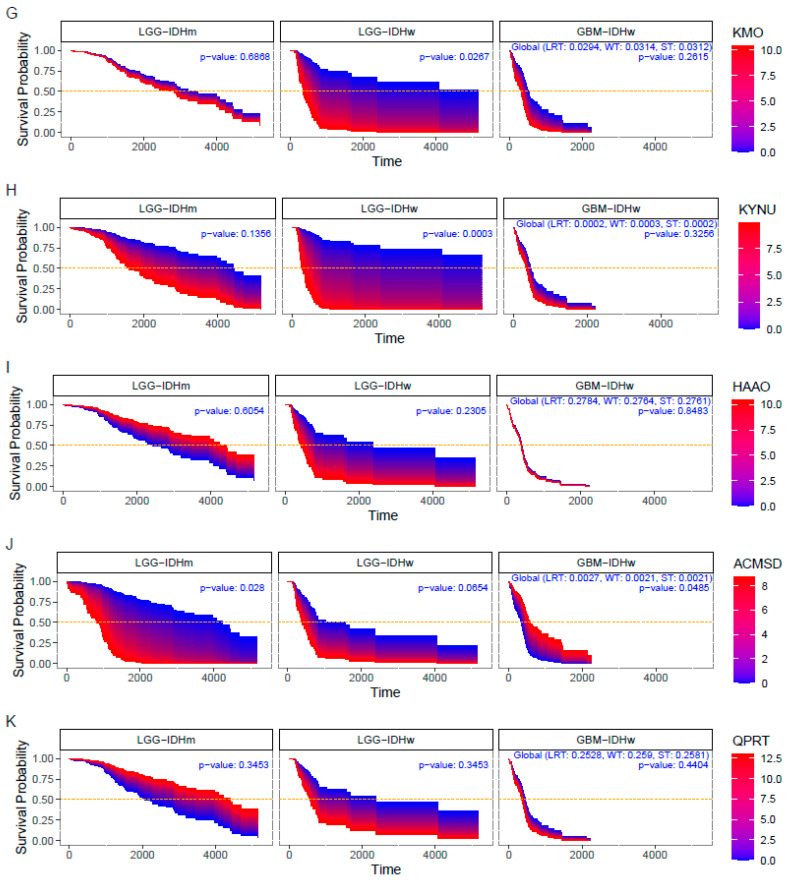
Survival area plots for patients diagnosed with IDHw low-grade glioma (LGG-IDHw), IDHm low-grade glioma (LGG-IDHm), and glioblastoma (GBM-IDHw) according to the expression levels of the kynurenine pathway enzymes. Survival probability of IDO1 (**A**), IDO2 (**B**), TDO2 (**C**), AFMID (**D**), AADAT (**E**), GOT2 (**F**), KMO (**G**), KYNU (**H**), HAAO (**I**), ACMSD (**J**), QPRT (**K**). For each KP enzyme, the associated Survival Probability curves are obtained from a Cox proportional hazards regression model.

Finally, it was explored if the expression of KP enzymes impacts on the patient survival among the two groups: IDHw/m LGG and IDHw GBM (Figure 3). We found that IDHw GBM patient survival was not affected by the expression of the KP enzymes, except the ACMSD (*p* = 0.0485). However, in IDHw LGG, the higher expression levels of IDO1 (*p* = 0.03), KMO (*p* = 0.02), and KYNU (*p* < 0.01) impacted negatively on patient survival, while the lower expression levels of AADAT (*p* = 0.04) were associated with lower survival rates. ACMSD (*p* = 0.02) was the only KP enzyme which presented a significant impact on the survival of IDHm LGG patients.

## 4. Discussion

In the CNS, the KP plays an important role due to the production of the neuroactive and redox metabolites involved in the redox balance, cognitive process, and social behavior [41,42,43]. However, it has been proposed that the KP metabolites could help malignant cells to overcome systemic barriers such as anti-tumor immune responses on several cancer types, including those of the CNS [44,45]. In this regard, Trp degradation by IDO and TDO had been the most extensively studied steps of the KP in cancer models, positioning the IDO expression as an immune checkpoint enzyme that abrogates the tumoral immune destruction [29,30,46]. This evidence drove the study of the IDO and TDO pharmacological inhibitors as anti-tumor therapeutic strategies; however, the use of these inhibitors has not been successful in clinical trials for GBM patients [47,48]. In recent years, the importance of other components of the KP downstream IDO and TDO have been studied on different cancer models including gliomas, where the expression of other enzymes such as KMO has been reported [32,33,34,35]. Because of the immunomodulatory properties of the molecules produced through the KP and bioenergetic relevance of this pathway as the source for the de novo synthesis of NAD+, in this study we were focused on the expression of the KP components and their relationship with the gene expressions related to the immune suppression, energy production, angiogenesis, and cell cycle disruption considering mainly three groups: LGG IDHw, LGG IDHm, and GBM. The classification depending on the IDH state was considered since it has been demonstrated that the IDH mutation promotes metabolic reprogramming that has been associated with a better prognosis [49]. In addition, recently it was demonstrated that the metabolic shift due to the IDH mutation increased the production of the oncometabolite D-2-hydroxyglutarate (D-2-HG), which inhibits the NAD+ biosynthesis [50,51].

As it was mentioned before, IDO and TDO start the Trp degradation and generate KYN. In recent years, a close relationship has been established between the expression of these enzymes and immunosuppression, leading to the study of IDO and TDO inhibition [47,48]. Although the use of IDO/TDO inhibitors by themselves has not been conclusive in clinical trials, their combination with immune checkpoint inhibitors has shown some effectiveness, which demonstrates the relevance of this pathway in the modulation of the anti-tumor immune system. Supporting previous studies, herein the IDO showed a high expression in all the CNS tumors which were analyzed compared with the non-tumor brain tissue independently of the IDH mutational status in gliomas. Several studies have described that the overexpression of IDO is associated with immune suppression, suggesting that the expression of this enzyme could modulate the expression of regulatory molecules of the immune system through non-enzymatic effects. Consistent with this idea, our data show a positive correlation between the IDO and inhibitory molecules of the innate immune response (HLAE and HLAG), as well as with the expression of immune checkpoint molecules (PD1 PDL1, CTLA4 y TIGIT). Additionally, the IDO expression was positively correlated with anti-inflammatory cytokines, and also with INF-γ, which is a known modulator of KP. The IL-2RA, a receptor constitutively expressed molecule in Treg, was correlated with the IDO expression.

On the other hand, it has been described that gliomas are highly vascularized and that up to 30% of these tumors are infiltrated with tumor-associated macrophages, which are known to participate in angiogenesis and produce high levels of metalloproteases. In concordance, the infiltrate as well as the metalloproteases MMP2 and MMP9 were positively correlated with the IDO expression. Additionally, the IDO expression was positively correlated with the signal transducer and activator of the transcription genes (STATs) [52]. The STATs are involved in the cellular cycle regulation, cellular survival, and the immune response. Specifically, STAT1 and STAT3, those which are IDO correlated, regulate the NK cells maturation, the anti-tumoral activity, as well as modulate the signaling mediate by the interferons type 1 and 2 [53,54]. These STATs are regulated by the intratumoral redox state, and in this sense, IDO1 was negatively correlated with the MTND1, NDUFS1, and SDHA expression, all genes involved in the oxidative phosphorylation. Taking into consideration the correlations between the IDO and gene set, it is possible to suggest that the IDO can favor or modulate molecules that negatively impact the global survival of patients with glioma.

Regarding IDO2, there are few reports about the participation of this enzyme in gliomas. It has been reported that this enzyme is apparently involved in the KYN-AhR-AQP4 signaling route, however, the mechanism of its participation is still unclear [46]. In this study, the IDO2 expression showed no differences between the gliomas and cerebral cortex and no significant associations were found with molecules related to the immune system, angiogenesis, cell cycle, GTPasas, or electron transport, results that are according to our findings.

Tryptophan 2,3-dioxygenase (TDO) was overexpressed in all the CNS tumors which were evaluated, suggesting a rapid degradation of TRP to KYN that causes Trp starvation in the tumor microenvironment and, consequently, the inhibition of the lymphocyte proliferation [55]. Specifically, in GBM, TDO was positively correlated with the expression of immunosuppressive molecules related with the evasion of innate immunity, checkpoint inhibitors, the angiogenic expression of VEGF, and the protease migration-related protein matrix metalloproteinase 9 (MMP9); TDO was negatively correlated with EGFR. The major contribution of TDO has been related to the immunosuppressive effects of KYN. KYN is a natural endogenous ligand of the human aryl hydrocarbon receptor (AHR) which activates the KYN-AhR-AQP4 signaling pathway supporting tumor growth and glioma cell motility [46], as well as the agonistic activity in the AHR leading to the differentiation onaïveaive to Treg lymphocytes [56]. In addition, the fact that KYN can be rapidly metabolized to other KP metabolites could be associated with TDOs poor impact on patient survival, as this study shows [31].

In addition to IDO and TDO, the AFMID is also involved in the KYN production. This enzyme has been poorly studied in cancer, however, a recent report indicated that the proto-oncogen MYC induces AFMID expression in colon cancer, and it is associated with cell proliferation through an AhR modulation by KYN [57]. In our study, this enzyme is down expressed in gliomas and P&P, while it is overexpressed in neuroblastoma. However, AFMID was not associated with the gene sets evaluated here, nor was an impact was found on the survival, suggesting that the principal KP enzymes involved in the KYN production on gliomas could be attributed to IDO and TDO.

Besides this, it was shown that the AADAT and GOT2 enzymes that represent the ‘short branch’ of the KP leading to the KYNA formation presented expression levels below non-tumor tissue. The AADAT and GOT2 expression favor competition for the substrate KYN, promoting the KYNA production, which has been implicated on the differentiation of Treg [58]. Interestingly, lower expression levels of AADAT corresponded to lower survival times and even more AADAT was the KP enzyme which showed negative correlations with the gene sets analyzed here. The low expression of this enzyme in tumor tissue could promote the degradation of KYN by KMO and KYNU, thus producing the KP-derived metabolites 3-HK and 3-HANA that have immunosuppressive functions [59]. KMO is the rate limiting enzyme of the KP long branch that takes KYN as a substrate to produce 3-HK. This enzyme is not expressed on healthy astrocytes; however, a recent report shows that glioma cells can express it [35]. Recent studies showed that KMO has a prognostic value on triple negative breast cancer, mainly on the recurrence and metastasis [32]. According to these findings KMO in addition to its enzymatic activity can reduce the kinase activity of GSK3β, which decreased the phosphorylation of β-catenin, making it susceptible to ubiquitination and posterior degradation [32]. Herein, KMO shows positive correlations with the immune response-related genes, and in the case of LGG-IDHw, a high expression of KMO was correlated with a poor survival.

On the other hand, the expression of the KYNU, HAAO, ACSMD, and QPRT enzymes, that constitute the ‘long branch’ of the KP and drives to NAD+ synthesis, were increased in the LLG IDHw and GBM compared to the brain cortex, which could promote the production of toxic molecules for anti-tumor immune populations [26,27] and support the correlation of the KP enzymes with tumor malignancy. It is of note that survival curves showed a worst prognostic for both IDHw and IDHm lower-grade gliomas when the KP enzymes, specifically ACSMD and KYNU, are overexpressed, indicating that KP-derived metabolites might be an effective marker of the outcome prediction in both lower- and high-grade gliomas.

Particularly, the KYNU expression showed positive correlations with the distinct gene sets related with the immune modulation, induction of angiogenesis, and enhanced proliferative signaling. The role of the KYNU on brain tumors remains poorly understood, however, its importance in different types of cancer has been revealed. In this regard, differences in the KYNU expression and activity within the triple negative and HER2-enriched breast cancer samples have been reported and a predictive model for triple negative and non-triple negative breast cancer diagnosis has been proposed based on the KYNU activity markers measured on the patient serum [60]. An additional study reported that the KYNU expression is negatively related with the HER2 and Ki67 levels on the breast cancer samples and positively related with the breast cancer differentiation stage [61]. In this way, the induced overexpression of KYNU on malignant cells suppressed the proliferation and tumor growth [61]. Besides this, the KYNU upregulation due to the constitutive activation of the redox regulator, the nuclear factor erythroid 2-related factor (Nrf2) was associated with immunosuppressive phenotypes and a lower survival of lung adenocarcinoma patients [62], while the KYNU knockdown decreased the malignant cell proliferation and migration through the downregulation of PI3K/AKT signaling on cutaneous squamous cell carcinoma [63].

In addition, the 3-HAAO and QPRT overexpression led gliomas to radiochemotherapy endurance via a resistance to oxidative stress by the overproduction of NAD+ [64]. It is important to note that the expression of these enzymes differs significantly (*p* < 0.0001) between the LGG IDHw and LGG IDHm, with the first showing the highest values, but it is of note that the estimation of the survival probability is completely inverse because in LGG IDHw, an overexpression of HAAO and QPRT has a worse prognosis, where in LGG IDHw, this has a better prognosis. This is in contrast to the fact that IDH mutation lead to the reprogram of the cellular metabolism that lead to a slow-growing feature and a high production of the oncometabolite D-2-HG that inhibits the biosynthesis of NAD+ by the Preiss–Handler pathway [50]. In that line, we should expect that the low production of NAD+ through the KP increase to be deleterious for glioma cells by the depletion of NAD+, but we found that this is not entirely clear because the overexpression of “long-branch enzymes” that lead to the NAD+ synthesis had a better prognosis in LGG IDHm, so more studies are required to understand why this inversion occurs.

As for the genes of the “hallmarks”, we found that 3-HAO is the second enzyme with more positive correlations in the gene set studies here, but it is of note that the lack of this enzyme is reported to non-neoplastic astrocytes and glioma cells, being the microglia of the principal cells where the 3-HAO is expressed to produce quinolinic acid, so then the glioma cells with an expression of QPRT can convert quinolinic acid to NAD+ [64]. However, another possibility is that the expression of 3-HAO in the bulk mass could come from tumor-infiltrating cells, like tumor-associated macrophages that have been reported as the main cell type that infiltrate glioblastoma tumors [65,66].

Nowadays, bioinformatic analysis represents a powerful tool for supporting or even for the propose of developing new research strategies among several life sciences disciplines. In this sense, bioinformatics helps us to understand the genotypic and phenotypic profiles and to design specific molecules that could be used as promising drugs. Particularly, in this study it was possible to explore the complex interactions between the impact of the KP enzymes and tumor microenvironment. Artificial intelligence, biotechnology, and nanoengineering are potential devices to design innovative peptide materials that could be used to design new drugs with a broad cellular penetration power to silencing KP enzymes; an excellent option could be cell-penetrating peptide amphiphiles, molecules with a capacity to form complexes with siRNAs, as an alternative for therapeutic delivery in glioma cells [67].

It is important to take into account that the high intratumoral heterogeneity within the tumor mass is very complex and that there are multiple factors involved in tumor development which could affect the estimates made in this study, but also it is important to keep in mind that the differences in the estimation could be dependent by the metabolic status of the sample at the time of the analysis. However, it is necessary to perform additional studies to clarify the impact of KP-related enzymes beyond IDO1.

## 5. Conclusions

Based on this study, the participation of components of the kynurenine pathway downstream of IDO1/TDO is highlighted, since they may be potential therapeutic targets that contribute to immunotherapy in cancer. Future studies should be focused on characterizing the participation of VK metabolites in the modulation of the immune response in order to understand their role in cancer.

## Figures and Tables

**Table 1:** Genes of KP enzymes and cancer hallmarks.

Grouping	Name	Abbreviation
Neoplasias	Glioblastoma	GBM
	Low-grade gliomas	LGG
	Low-grade glioma with IDH-1 mutation	LLG IDHm
	Low-grade glioma with IDH-1 wildtype	LLG IDHw
	Glioblastoma multiforme with IDH-1 wildtype	GBM IDHw
	Glioblastoma multiforme with IDH-1 mutation	GBM IDHm
Kynurenine pathway	Tryptophan dioxygenase	TDO
	Indoleamine dioxygenase	IDO
	Arylformamidase	AFMID
	Glutamic-oxaloacetic transaminase	GOT2
	Aminoadipate aminotransferase	AADAT
	Kynureninase	KYNU
	Kynurenine monooxygenase	KMO
	Quinolinic acid phosphoribosyl transferase	QPRT
	3-HANA dioxygenase	HAAO
	Aminocarboxymuconate semialdehyde decarboxylase	ACMSD
Immune response	Human leukocyte antigen E	HLA-E
	Human leukocyte antigen G	HLA-G
	Programmed cell death 1	PD-1/CD274
	Programmed cell death 1 ligand 2	PDCD1LG2
	Cytotoxic T lymphocyte antigen 4	CTLA4
	T cell immunoreceptor with Ig and ITIM domains	TIGIT
	Hepatitis A virus cellular receptor 2	HAVCR2
	Lymphocyte activating 3	LAG3
	Interferon gamma	IFNG
	Tumor necrosis factor	TNF
	Transforming growth factor beta 1	TGFB1
	Interleukin 1 beta	IL1B
	Interleukin 6	IL6
	Interleukin 10	IL10
	Interleukin 13	IL13
	Interleukin 13 receptor subunit alpha 2	IL13RA2
	Interleukin 2 receptor subunit alpha	IL2RA
Angiogenesis	Vascular endothelial growth factor	VEGF
	Vascular endothelial growth factor A	VEGFA
	Vascular endothelial growth factor B	VEGFB
	Vascular endothelial growth factor C	VEGFC
	Matrix metalloproteinase 2	MMP2
	Matrix metalloproteinase 9	MMP9
	Fibroblast growth factor 1	FGF1
	Fibroblast growth factor 2	FGF2
	Fibroblast growth factor receptor 1	FGFR1
	Hepatocyte growth factor	HGF
	Epidermal growth factor receptor	EGFR
	Phosphatase and tensin homolog	PTEN
Signal transducer and activator of transcription (STAT)	Signal transducer and activator of transcription 1	STAT1
	Signal transducer and activator of transcription 2	STAT2
	Signal transducer and activator of transcription 3	STAT3
	Signal transducer and activator of transcription 4	STAT4
Rho GTPases	Cell division control protein 42 homolog	CDC42
	Rac family small GTPase 1	RAC1
	Ras homolog family member A	RHOA
Electron transport chain: complexes I and II	Mitochondrially encoded NADH:ubiquinone oxidoreductase core subunit 1	MT-ND1
	NADH:ubiquinone oxidoreductase core subunit S1	NDUFS1
	Succinate dehydrogenase complex flavoprotein subunit A	SDHA
	Succinate dehydrogenase complex iron sulfur subunit B	SDHB

## Data Availability

The data presented in this study are openly available in UCSC Xena. at https://doi.org/10.1038/s41587-020-0546-8 (accessed on 1 September 2022).

## References

[1] Cahill D., Turcan S. (2018). Origin of Gliomas. Semin. Neurol..

[2] Louis D.N., Perry A., Wesseling P., Brat D.J., A Cree I., Figarella-Branger D., Hawkins C., Ng H.K., Pfister S.M., Reifenberger G. (2021). The 2021 WHO Classification of Tumors of the Central Nervous System: A summary. Neuro Oncol..

[3] Stupp R., Brada M., van de Bent M.J., Tonn J.-C., Pentheroudakis G. (2014). High-grade glioma: ESMO Clinical Practice Guidelines for diagnosis, treatment and follow-up. Ann. Oncol..

[4] Stupp R., Mason W.P., van de Bent M.J., Weller M., Fisher B., Taphoorn M.J.B., Belanger K., Brandes A.A., Marosi C., Bogdahn U. (2005). Radiotherapy plus concomitant and adjuvant temozolomide for glioblastoma. N. Engl. J. Med..

[5] Ostrom Q.T., Patil N., Cioffi G., Waite K., Kruchko C., Barnholtz-Sloan J.S. (2020). CBTRUS Statistical Report: Primary Brain and Other Central Nervous System Tumors Diagnosed in the United States in 2013-2017. Neuro. Oncol..

[6] Hanahan D., Weinberg R.A. (2011). Hallmarks of cancer: The next generation. Cell.

[7] Fouad Y.A., Aanei C. (2017). Revisiting the hallmarks of cancer. Am. J. Cancer Res..

[8] Negrini S., Gorgoulis V.G., Halazonetis T.D. (2010). Genomic instability--an evolving hallmark of cancer. Nat. Rev. Mol. Cell Biol..

[9] Verhaak R.G., Hoadley K.A., Purdom E., Wang V., Yuan Q., Wilkerson M.D., Miller C.R., Ding L., Golub T., Mesirov J.P. (2010). Integrated genomic analysis identifies clinically relevant subtypes of glioblastoma characterized by abnormalities in PDGFRA, IDH1, EGFR, and NF1. Cancer Cell.

[10] Ohgaki H., Kleihues P. (2013). The definition of primary and secondary glioblastoma. Clin. Cancer Res..

[11] Agnihotri S., Zadeh G. (2016). Metabolic reprogramming in glioblastoma: The influence of cancer metabolism on epigenetics and unanswered questions. Neuro Oncol..

[12] Liberti M.V., Locasale J.W. (2016). The Warburg Effect: How Does it Benefit Cancer Cells?. Trends Biochem. Sci..

[13] Strickland M., Stoll E.A. (2017). Metabolic Reprogramming in Glioma. Front. Cell Dev. Biol..

[14] Shibao S., Minami N., Koike N., Niboyuki F., Yoshida K., Saya H., Sampetrean O. (2018). Metabolic heterogeneity and plasticity of glioma stem cells in a mouse glioblastoma model. Neuro Oncol..

[15] Vlashi E., Lagadec C., Vergnes L., Matsutani T., Masui K., Poulou M., Popescu R., Donna L.D., Evers P., Dekmezian C. (2011). Metabolic state of glioma stem cells and nontumorigenic cells. Proc. Natl. Acad. Sci. USA.

[16] Ye F., Zhang Y., Liu Y., Yamada K., Tso J.L., Menjivar J.C., Tian J.Y., Yong W.H., Schaue D., Mischel P.S. (2013). Protective properties of radio-chemoresistant glioblastoma stem cell clones are associated with metabolic adaptation to reduced glucose dependence. PLoS ONE.

[17] Saga I., Shibao S., Okubo J., Osuka S., Kobayashi Y., Yamada S., Fujita S., Urakami K., Kusuhara M., Yoshida K. (2014). Integrated analysis identifies different metabolic signatures for tumor-initiating cells in a murine glioblastoma model. Neuro Oncol..

[18] Kim J., Han J., Jang Y., Kim S.J., Lee M.J., Ryu M.J., Kweon G.R., Heo J.Y. (2015). High-capacity glycolytic and mitochondrial oxidative metabolisms mediate the growth ability of glioblastoma. Int. J. Oncol..

[19] Waziri A. (2010). Glioblastoma-derived mechanisms of systemic immunosuppression. Neurosurg. Clin. N. Am..

[20] Broekman M.L., Mass S.L.N., Abels E.R., Mempel T.R., Krichevsky A.M., Breakefield X.O. (2018). Multidimensional communication in the microenvirons of glioblastoma. Nat. Rev. Neurol..

[21] Gieryng A., Pszczolkowska D., Walentynowicz K.A., Rajan W.D., Kaminska B. (2017). Immune microenvironment of gliomas. Lab. Investig..

[22] Perng P., Lim M. (2015). Immunosuppressive Mechanisms of Malignant Gliomas: Parallels at Non-CNS Sites. Front. Oncol..

[23] Badawy A.A. (2017). Tryptophan availability for kynurenine pathway metabolism across the life span: Control mechanisms and focus on aging, exercise, diet and nutritional supplements. Neuropharmacology.

[24] Bender D.A., Magboul B.I., Wynick D. (1982). Probable mechanisms of regulation of the utilization of dietary tryptophan, nicotinamide and nicotinic acid as precursors of nicotinamide nucleotides in the rat. Br. J. Nutr..

[25] Gossmann T.I., Ziegler M., Puntervoll P., de Figueiredo L.F., Schuster S., Heiland I. (2012). NAD(+) biosynthesis and salvage--a phylogenetic perspective. FEBS J..

[26] Frumento G., Rotondo R., Tonetti M., Damonte G., Benatti U., Ferrara G.B. (2002). Tryptophan-derived catabolites are responsible for inhibition of T and natural killer cell proliferation induced by indoleamine 2,3-dioxygenase. J. Exp. Med..

[27] Belladonna M.L., Puccetti P., Orabona C., Fallarino F., Vacca C., Volpi C., Gizzi S., Pallotta M.T., Fioretti M.C., Grohmann U. (2007). Immunosuppression via tryptophan catabolism: The role of kynurenine pathway enzymes. Transplantation.

[28] Tanaka M., Tóth F., Polyák H., Szabó A., Mándi Y., Vécsei L. (2021). Immune Influencers in Action: Metabolites and Enzymes of the Tryptophan-Kynurenine Metabolic Pathway. Biomedicines.

[29] Kudo T., Prentzell M.T., Mohapatra S.R., Sahm F., Zhao Z., Grummt I., Wick W., Opitz C.A., Platten M., Green E.W. (2020). Constitutive Expression of the Immunosuppressive Tryptophan Dioxygenase TDO2 in Glioblastoma Is Driven by the Transcription Factor C/EBPbeta. Front. Immunol..

[30] Mohapatra S.R., Sadik A., Tykocinski L.O., Dietze J., Poschet G., Heiland I., Opitz C.A. (2019). Hypoxia Inducible Factor 1alpha Inhibits the Expression of Immunosuppressive Tryptophan-2,3-Dioxygenase in Glioblastoma. Front. Immunol..

[31] Wainwright D.A., Balyasnikova I.V., Chang A.L., Ahmed A.U., Moon K.S., Auffinger B., Tobias A.L., Han Y., Lesniak M.S. (2012). IDO expression in brain tumors increases the recruitment of regulatory T cells and negatively impacts survival. Clin. Cancer Res..

[32] Huang T.T., Tseng L.M., Chen J.L., Chu P.Y., Lee C.H., Huang C.T., Wang W.L., Lau K.Y., Tseng M.F., Chang Y.Y. (2020). Kynurenine 3-monooxygenase upregulates pluripotent genes through beta-catenin and promotes triple-negative breast cancer progression. EBioMedicine.

[33] Liu C.Y., Huang T.T., Chen J.l., Chu P.Y., Lee C.H., Lee H.C., Lee Y.H., Chang Y.Y., Yang S.H., Jiang J.K. (2021). Significance of Kynurenine 3-Monooxygenase Expression in Colorectal Cancer. Front. Oncol..

[34] Liu I.L., Chung T.F., Huang W.H., Hsu C.H., Liu C.C., Chiu Y.Y., Huang K.C., Liao A.T.C., Chen S.L. (2021). Kynurenine 3-monooxygenase (KMO), and signal transducer and activator of transcription 3 (STAT3) expression is involved in tumour proliferation and predicts poor survival in canine melanoma. Vet. Comp. Oncol..

[35] Vazquez Cervantes G.I., Pineda B., Ramírez Ortega D., Salazar A., González Esquivel D.F., Rembao D., Zavala Vega S., Gómez Manzo S., Pérez de la Cruz G., Pérez de la Cruz V. (2021). Kynurenine Monooxygenase Expression and Activity in Human Astrocytomas. Cells.

[36] Goldman M.J., Craft B., Hastie M., Repecka K., McDade F., Kamath A., Banerjee A., Yunhai L., Rodgers D., Brooks A.N. (2020). Visualizing and interpreting cancer genomics data via the Xena platform. Nat. Biotechnol..

[37] R Core Team (2020). R: A Language and Environment for Statistical Computing. R Foundation for Statistical Computing, Vienna. https://www.r-project.org.

[38] Therneau T.M. (2022). A Package for Survival Analysis in R. R package version 3.4-0. https://CRAN.R-project.org/package=survival.

[39] Hothorn T., Bretz F., Westfall P. (2008). Simultaneous inference in general parametric models. Biom J..

[40] Revelle W. (2019). *psych: Procedures for Psychological, Psychometric, and Personality Research*. R Package Version 1.9.12, Northwestern University. https://CRAN.R-project.org/package=psych.

[41] Gonzalez Esquivel D., Ramirez Ortega D., Pineda B., Castro N., Rios C., Pérez de la Cruz V. (2017). Kynurenine pathway metabolites and enzymes involved in redox reactions. Neuropharmacology.

[42] Ramirez Ortega D., Ovalle Rodríguez P., Pineda B., González-Esquivel D.F., Ramos Chávez L.A., Vazquez-Cervantes G.I., Roldán-Roldán G., Pérez de la Cruz G., Díaz Ruiz A., Méndez Armenta M. (2020). Kynurenine Pathway as a New Target of Cognitive Impairment Induced by Lead Toxicity During the Lactation. Sci. Rep..

[43] Ramos-Chavez L.A., Roldán-Roldán G., García-Juárez B., González-Esquivel D., Pérez de la Cruz V., Pineda B., Ramírez- Ortega D., García Muñoz I., Jímenez Herrera B., Ríos C. (2018). Low Serum Tryptophan Levels as an Indicator of Global Cognitive Performance in Nondemented Women over 50 Years of Age. Oxid. Med. Cell Longev..

[44] Prendergast G.C. (2011). Cancer: Why tumours eat tryptophan. Nature.

[45] Adams S., Braidy N., Bessede A., Brew B.J., Grant R., Teo C., Guillemin G.J. (2012). The kynurenine pathway in brain tumor pathogenesis. Cancer Res..

[46] Du L., Xing Z., Tao B., Li T., Yang D., Li W., Zheng Y., Kuang C., Yang Q. (2020). Both IDO1 and TDO contribute to the malignancy of gliomas via the Kyn-AhR-AQP4 signaling pathway. Signal. Transduct. Target Ther..

[47] Wainwright D.A., Chang A.L., Dey M., Balyasnikova I.V., Kim C.-K., Tobias A., Cheng Y., Kim J.W., Qiao J., Zhang L. (2014). Durable therapeutic efficacy utilizing combinatorial blockade against IDO, CTLA-4, and PD-L1 in mice with brain tumors. Clin. Cancer Res..

[48] Hanihara M., Oh-Oka K., Mitsuka K., Nakao A., Kinouchi H. (2016). Synergistic antitumor effect with indoleamine 2,3-dioxygenase inhibition and temozolomide in a murine glioma model. J. Neurosurg..

[49] Eckel-Passow J.E., Lachance D.H., Molinaro A.M., Walsh K.M., Decker P.A., Sicotte H., Pekmezi M., Rice T., Kosel M.T., Smirnov I.V. (2015). Glioma Groups Based on 1p/19q, IDH, and TERT Promoter Mutations in Tumors. N. Engl. J. Med..

[50] Tateishi K., Wakimoto H., Lafrate A.J., Tanaka S., Loebel F., Lelic N., Wiederschain D., Bedel O., Deng G., Zhang B. (2015). Extreme Vulnerability of IDH1 Mutant Cancers to NAD+ Depletion. Cancer Cell.

[51] Han S., Liu Y., Cai S.J., Qian M., Ding J., Larion M., Gilbert M.R., Yang C. (2020). IDH mutation in glioma: Molecular mechanisms and potential therapeutic targets. Br. J. Cancer.

[52] Butturini E., de Prati A.C., Mariotto S. (2020). Redox Regulation of STAT1 and STAT3 Signaling. Int. J. Mol. Sci..

[53] Meissl K., Simonovic N., Amenitsch L., Witalisz-Siepracka A., Klein K., Lassnig C., Puga A., Vogl C., Poelzl A., Bosmann M. (2020). STAT1 Isoforms Differentially Regulate NK Cell Maturation and Anti-tumor Activity. Front. Immunol..

[54] Goder A., Ginter T., Heinzel T., Stroh S., Fahrer J., Henke A., Kramer O.H. (2021). STAT1 N-terminal domain discriminatively controls type I and type II IFN signaling. Cytokine.

[55] Yu C.P., Pan Z.Z., Luo D.Y. (2016). TDO as a therapeutic target in brain diseases. Metab. Brain Dis..

[56] Walczak K., Wnorowski A., Turski W.A., Plech T. (2020). Kynurenic acid and cancer: Facts and controversies. Cell. Mol. Life Sci..

[57] Venkateswaran N., Lafita-Navarro M.C., Hao Y., Kilgore J.A., Perez-Castro L., Braverman J., Borenstein-Auerbach N., Kim M., Lesner N.P., Mishra P. (2019). MYC promotes tryptophan uptake and metabolism by the kynurenine pathway in colon cancer. Genes Dev..

[58] Mezrich J.D., Fechner J.H., Zhang X., Jhonson B.P., Burlingham W.J., Bradfield C.A. (2010). An interaction between kynurenine and the aryl hydrocarbon receptor can generate regulatory T cells. J. Immunol..

[59] Kesarwani P., Kant S., Prabhu A., Chinnaiyan P. (2017). The interplay between metabolic remodeling and immune regulation in glioblastoma. Neuro Oncol..

[60] Heng B., Bilgin A.A., Lovejoy D.B., Tan V.X., Milioli H.H., Gluch L., Bustamante S., Sabaretnam T., Moscato P., Lim C.K. (2020). Differential kynurenine pathway metabolism in highly metastatic aggressive breast cancer subtypes: Beyond IDO1-induced immunosuppression. Breast Cancer Res..

[61] Liu Y., Feng X., Lai J., Yi W., Yang J., Du T., Long X., Zhang Y., Xiao Y. (2019). A novel role of kynureninase in the growth control of breast cancer cells and its relationships with breast cancer. J. Cell. Mol. Med..

[62] Fahrmann J.F., Tanaka I., Irajizad E., Mao X., Dennison J.B., Murage E., Casabar J., Mayo J., Peng Q., Celiktas M. (2022). Mutational Activation of the NRF2 Pathway Upregulates Kynureninase Resulting in Tumor Immunosuppression and Poor Outcome in Lung Adenocarcinoma. Cancers.

[63] Ci C., Wu C., Lyu D., Chang X., He C., Liu W., Chen L. (2020). Downregulation of kynureninase restrains cutaneous squamous cell carcinoma proliferation and represses the PI3K/AKT pathway. Clin. Exp. Dermatol..

[64] Sahm F., Oezen I., Optiz C.A., Radlwimmer B., von Deimling A., Ahrendt T., Adams S., Bode H.B., Guillemin G.G., Wick W. (2013). The endogenous tryptophan metabolite and NAD+ precursor quinolinic acid confers resistance of gliomas to oxidative stress. Cancer Res..

[65] Tang M., Xie Q., Gimple R.C., Zhong Z., Tam T., Tian J., Kidwell R.L., Wu Q., Praguer B.C., Qiu Z. (2020). Three-dimensional bioprinted glioblastoma microenvironments model cellular dependencies and immune interactions. Cell Res..

[66] Fu W., Wang W., Li H., Jiao Y., Huo R., Zihan Y., Wang J., Wang S., Wang J., Chen D. (2020). Single-Cell Atlas Reveals Complexity of the Immunosuppressive Microenvironment of Initial and Recurrent Glioblastoma. Front. Immunol..

[67] Feger G., Angelov B., Angelova A. (2020). Prediction of Amphiphilic Cell-Penetrating Peptide Building Blocks from Protein-Derived Amino Acid Sequences for Engineering of Drug Delivery Nanoassemblies. J. Phys. Chem. B.

